# A Culture of Health and Alcohol-Permitted Events at a U.S. University

**DOI:** 10.1007/s10935-022-00686-z

**Published:** 2022-06-13

**Authors:** Tanya Nieri, Megan Webb, Deja Goodwin, Min Yoo

**Affiliations:** 1 Sociology Department, University of California at Riverside, Watkins 1214, Riverside, CA 92521 USA; 2 Sociology Department, UC Los Angeles, 264 Haines Hall, Los Angeles, CA 90095-1551 USA; 3 Sociology Department, Loyola Marymount University, 1 LMU Drive, Los Angeles, CA 90045 USA

**Keywords:** Culture of health, Healthy campus, Alcohol culture, Environmental prevention

## Abstract

We examined event organizers’ understandings and management of alcohol-related risk and accommodation of people in recovery from substance use disorders and other non-drinkers, when organizing alcohol-permitted events that primarily involved faculty, staff, and graduate students. We interviewed 31 event organizers at a large, public university in California. Organizers were most concerned about avoiding legal liabilities, were less concerned about promoting responsible drinking among drinkers, and often failed to consider the needs of non-drinkers. Their actions were informed by problematic beliefs about alcohol (e.g., people need alcohol to relax and socialize), drinkers (e.g., only undergraduate students engage in risky alcohol consumption), and people in recovery (e.g., they lack self-control). Organizers over-relied on informal control to shape attendees’ behavior, failing to acknowledge contextual factors. They need education on how they can shape the event context to better promote healthy behaviors, avoid exclusively focusing on informal control and prevention of unhealthy behaviors, and promote better inclusion of people who do not drink alcohol. There is fertile ground for infusing a culture of health into events in higher education.

**Supplementary Information:**

The online version contains supplementary material available at 10.1007/s10935-022-00686-z.

## Background

### Healthy Campus

Higher education institutions in the United States aim to promote the health of students and employees through separate measures (e.g., wellness centers for students and employee health benefits for employees) (American College Health Association, 2018a). They have recently begun to treat faculty, staff, and students, and even residents of the surrounding geographic area, as part of a common group and consider how the institution’s daily operations affect the health of community members. The emerging Healthy Campus movement emphasizes prevention and draws on social ecological models of health, which acknowledge factors beyond the individual (e.g., institutions, built environment, policies) that affect individual health (Sallis & Owen, 2015), and research documenting how community characteristics affect community members’ health (e.g., Carpiano, 2007).

One Healthy Campus strategy is to promote at all levels of an institution a culture of health. Culture is “a set of practices and behaviours defined by customs, habits, language, and geography that groups of individuals share” (Napier et al., 2014, p. 1609). A culture of health, then, is a set of practices and behaviors that reflects health as a shared, rather than individual, value (Seifer, 2018). In a culture of health the university and its community members share responsibility for promoting health, and this shared responsibility is cultivated and supported by institutional organization and practices. Thus, the emphasis shifts from the individual to the university context or structure. For example, rather than focusing on individuals’ food choices via wellness programs, a culture-of-health approach focuses on the food options available on campus, aiming to provide healthy food options to enable healthy choices by individuals. Similarly, rather than focusing on individuals’ discriminatory behavior via student conduct or employee regulations, a culture-of-health approach focuses on the extent to which campus spaces and activities equitably include diverse community members. Considering campus events, a culture-of-health approach focuses on the extent to which events do more than avoid liability for the university. It aims to encourage events that promote health by providing a context in which healthy choices can be made and diverse community members are included. Given the increase in Healthy Campus initiatives (American College Health Association, 2018b), we need to better understand how a culture of health can be incorporated into existing institutional practices and structures.

### Environmental Prevention of Substance Use Harm

Research on substance use has documented not only event-related risks but also successful strategies for risk reduction at events, such as the Healthy Nightlife Toolbox (http://www.hntinfo.eu/). This literature documents the value of environmental prevention: population-based strategies that change the context in which people make decisions and have the capacity to affect large numbers of people (Freiden, 2010; McLeroy et al., 1988). This approach aims to reduce substance use harm (Oncioiu et al., 2018; Treno & Lee, 2002). Less is known about how these strategies might relate to non-drinkers’ experiences at events with alcohol.

### The Need to Broaden Research on Alcohol in Higher Education

Research on college substance use overwhelmingly focuses on undergraduates. It has documented how institutional characteristics, such as unclear or poorly accessible drinking policies (Jernigan et al., 2019), a party culture (Vander Ven, 2011), a large Greek system (Presley et al., 2002), specific campus events (Neighbors, et al., 2011), and popular sports teams (Neal & Fromm, 2007), contribute to the students’ harmful alcohol use. It has also investigated how institutions can address student use and associated harms—for example, by offering alcohol-free events (Layland et al., 2019) and employing environmental strategies (Saltz, 2011; Wolfson et al., 2012). This research documents the promise of institutional approaches that go beyond individual risk to address community factors (Saltz, 2011).

There is increased enrollment of college students who are in recovery from substance use disorders (Cleveland et al., 2007). While the exact percentage of students in recovery from substance use disorders among the 22.25 million recovering adults in the United States is unknown (Kelly et al., 2017), one in six students are estimated to meet the criteria for current alcohol abuse or dependence (Bugbee et al., 2016). College campuses have been characterized as “abstinence-hostile environments” that impede entry into and maintenance of recovery (Cleveland et al., 2007). Scholars have highlighted the need for research on this group of students (Perron et al., 2011) and begun to explore the ways in which higher education institutions can support it, such as through collegiate recovery programs (Cleveland et al., 2007; Laitman & Stewart, 2019).

We also need research on faculty and staff. The few, dated studies on these groups (Spickard & Billings, 1986; Watts et al., 1991) highlight how, among other risk factors, work conditions (e.g., level of stress, extent of supervision) can influence risk. While highlighting the value of individual-level approaches, such as employee assistance programs (Watts et al., 1991), this research also calls for recognition of alcoholism as an institutional health problem, one that the institutions can address (Spickard & Billings, 1986).

Finally, prior research on drinking culture in higher education has heavily focused on students’ private social sphere and has not examined culture in other spheres of university life, such as academic departments.

### The Need for Research on Alcohol-Permitted Events

Alcohol is commonly offered at work-related events, blurring the line between work and play (Gusfeld, 2003). In this context alcohol may be intended to serve a symbolic function, such as the way a toast to a retiring colleague signifies honor and respect for the colleague. It may also be intended to serve a practical function, such as guest recruitment, when the availability of alcohol is billed as a benefit of event attendance. Alcohol consumption is commonly viewed as a leisure activity and thus, can be perceived to add fun to a work-related event (Gusfeld, 2003; Peele & Grant, 1999). In fact, some people, especially young people, misperceive alcohol to be necessary for fun (Martinic & Measham, 2008; Vander Ven, 2011).

Faculty, staff, and students participate in alcohol-permitted events in their work roles, often achieving work-related ends (e.g., professional networking and team building). Accordingly, the university has an interest in preventing liability. One way that institutions manage alcohol-related risk is by issuing permits for events with alcohol. Permits aim to ensure that event organizers implement safety precautions, such as checking identification to enforce the drinking age minimum and training beverage servers to recognize, and refuse service to, intoxicated persons and mix drinks in accepted proportions. These precautions aim to prevent adverse events and protect the institution from liability (Heath & McCarty, 1995; Kumpfer, 2002). However, research has not explored the extent to which permits contribute to producing a culture of health at an event.

While scholars have documented the existence of drinking cultures (Aresi & Bloomfield, 2021; MacLean et al., 2020; Savic et al., 2016; Vander Ven, 2011), addiction culture (White, 1996) and recovery culture (Holleran & Macmaster, 2005; Snyder & Fessler, 2014), little research has examined the confluence of these cultures at university events. While strategies for addressing culture exist, little research has examined how to simultaneously accommodate the cultures of drinkers and non-drinkers. For example, social norms approaches to college prevention are geared toward people at risk of use, not people at risk of relapse (Perkins & Perkins, 2018). Campus events not geared toward people in recovery may undermine recovery. For example, reliance on informal behavioral controls may be ineffective for people with addictive disorders (White, 1996). The recovery literature highlights the importance, for people in recovery, of mobilizing support beyond professional treatment and raises the possibility that universities may serve as recovery support institutions (White, 2012).

### Aims and Contributions of the Present Study

This study aims to (1) describe event organizers’ understandings and management of alcohol-related risk and accommodation of people in recovery from substance use disorders and other non-drinkers, when organizing alcohol-permitted events that primarily involved faculty, staff, and graduate students, and (2) identify opportunities for deploying culture-of-health approaches to organizing events with alcohol.

The study makes several important contributions. First, the study adds to the literature on community health and Healthy Campus by examining whether and how event organizers employ a culture-of-health approach when planning alcohol-permitted events. Second, it addresses gaps in the literatures on environmental prevention and alcohol in higher education by examining events involving faculty, staff, and students, the inclusion of people in recovery from substance use disorders, the public sphere (i.e., workplace events), and institutional practices, specifically event planning. Third, the study fills gaps in research on alcohol-permitted events by examining event organizers’ understandings of how permits relate to alcohol-related risk reduction and health promotion. It explores the extent to which university event organizers aim to provide an event space that is supportive of, or at least not hostile toward, people in recovery. Finally, since university events may also include people who are abstinent for other reasons (e.g., health or religion), this study explores how event organizers consider abstainers when planning events.

## Methods

### Study Design

The study was conducted at a large, public university in California and approved by its Institutional Review Board. We obtained a copy of the university Risk Management Office’s database of alcohol-permitted events from 7/1/2018 to 6/30/2019. No-cost permits are required for university events with alcohol. They cannot be requested for undergraduate events. To get a permit, organizers provide information on the alcohol to be served, who will serve it (a licensed server or other), whether it will be free or for purchase, whether attendance is open or by invitation, the number and nature of participants, the availability of food, the event purpose, and the event location.

### Sample Selection

An analysis of the database informed our purposive selection of event organizers to interview. We employed a maximum variability sampling strategy, selecting “cases with maximum variation for the purpose of documenting unique or diverse variations that have emerged in adapting to different conditions, and to identify important common patterns that cut across variations” (Palinkas et al., 2015, p. 534). Thus, the criteria for capturing variability in the event characteristics were as follows: we included recurring events (which tended to be work-related such as seminar series) and one-time events (which tended to be social or celebratory such as holiday parties), events planned by different campus units, and events planned by different organizers: faculty, staff, or graduate students. We initially identified 55 different events organized by 42 people.

Once we selected the events, we contacted, by phone or email, the people listed as the event organizer in the permit database, using the contact information in the database. Seven organizers refused an interview (indicating lack of time) or were unreachable (did not reply to our inquiries). In 2019, we conducted in-person interviews with 31 people who organized 31 focal events (8 social events, 7 celebratory events, and 16 work events). We reached data saturation with these interviews, meaning new data were redundant and participant responses were similar for our research questions (Guest et al., 2017; Saunders et al., 2018) about how organizers understand and manage alcohol-related risk and accommodate people in recovery from substance use disorders and people who do not drink. Thus, the remaining four selected organizers were not interviewed. Table 1 provides participant information.

**Table 1:** Participant information

	Age	Gender	Job title	Number of events organized
Jack	35	Male	Faculty member	3
Sally	64	Female	Faculty member	1
Marion	31	Female	Faculty member	2
Madison	58	Female	Faculty member	19
Yuna	50 +	Female	Faculty member	2
Daniel	31	Male	Graduate student	1
Peter	33	Male	Graduate student	16
Jeremy	29	Male	Graduate student	1
Kayla	26	Female	Graduate student	4
Morgan	36	Female	Staff: Administrative Officer	35
Shelby	50	Female	Staff: Executive Assistant	1
Shannon	48	Female	Staff: Grant analyst	1
John	29	Male	Staff: Student Advisor	1
Elena	44	Female	Staff: Program director	1
Kate	55	Female	Staff: Executive Assistant	4
Evelyn	55	Female	Staff: Executive Administrator	1
Anthony	24	Male	Staff: Academic Analyst	5
Yasmin	29	Female	Staff: Program Coordinator	2
Isaac	35	Male	Staff: Development Director	1
Diane	35	Female	Staff: Executive Assistant	2
Brenda	50 +	Female	Staff: Assistant	1
Rianne	55	Female	Staff: Administrative Assistant	1
Carrie	33	Female	Staff: Manager	1
Taylor	59	Female	Staff: Executive Assistant	1
Christine	61	Female	Staff: Event Specialist	4
Jim	54	Male	Staff: Lab Supervisor	1
Penelope	56	Female	Staff: Administrative Assistant	3
Greg	23	Female	Staff: Events Coordinator	5
Anna	49	Female	Staff: Events Manager	1
Jane	–	Female	Staff: Administrative Coordinator	1
Sarah	–	Female	Staff: Financial analyst	1

### Procedures

In 2019, we contacted selected participants and indicated our interest in conducting a brief interview with them about the event(s) for which they had requested a permit(s). We clarified that their work performance was not being evaluated. The audio-recorded, one-time interviews were conducted by student research assistants who received training in interviewing technique. They occurred in the participants’ work setting (i.e., at their desk, in a conference room) and ranged in duration from 20–45 min, lasting on average 25 min.

### Instrument

Supplementary Table 1 contains the interview protocol.

### Data Analysis

Our analysis centered on identifying opportunities for intervention to better promote a culture of health at campus events, not on evaluating the permitting process. We conducted a descriptive analysis of the permit data to summarize the alcohol-permitted events that occurred in the review period. We coded for whether the event was one-time or part of a series, the type of alcohol served, whether the event was public or private, who was invited, the number of attendees, the timing of the event (part of the work day or after hours), the location of the event (on or off campus), and the type of event.

In addition to the descriptive analysis of permit data, we conducted a thematic analysis of the interview data. The first two authors read all transcripts and developed thematic codes to answer the following questions: What were the reasons alcohol was provided at the events?, How were alcohol-related risks managed and responsible drinking promoted?, and What accommodations were made for non-drinkers? These thematic questions were derived from the research aims. This coding strategy is in line with what Constas (1992) terms the “origination” component of data categorization in which thematic codes or categories are developed based on the interest of the researcher or the overarching interests of the research project. The themes that emerged from the data included the stated motivations for serving alcohol at the event (e.g., making the event more fun, alcohol needed for celebratory events, etc.), ideas about the risks associated with serving alcohol at campus events (e.g., if certain types of alcohol are more risky, or if the characteristics of attendees make serving alcohol more risky), strategies for managing alcohol-related risk (informal control, such as relying on individuals’ willpower, versus formal control), and the accommodations made for non-drinkers. The second author then coded based on these criteria and identified illustrative quotes. See Table 2 for the codes and sample quotations. Once codes were applied, we assessed whether there were differences by organizer: faculty, staff, and students. We found no differences between organizers; the set of codes that emerged for each type was similar and no one type of organizer was concentrated in any particular code.

**Table 2:** Thematic codes and sample quotations

Code	Sample quotation
*Motivations for having alcohol at event*	
Incentivize attendance	“…part of it was to incentivize people to stay for a reception”
Promote socializing, celebrating and fun	“It’s because of the holidays. Just, you know, just be festive”
Satisfy people’s want for alcohol	“…it’s what the faculty want”
Promote inclusion	“having it as an option for those that would like a drink is typically a little more inclusive than not offering it at all”
*Ideas about alcohol risk*	
Promotion of responsible drinking	“It hasn’t been (promoted)” “Just common sense”
Considerations of risk	“… it’s such a small event, it’s a lot easier to—we don’t have to worry as much”
*Alcohol risk management*	
Informal control	“…but they are all pretty much adults and they all pretty much know their limits”
Formal control	“…people who serve alcohol…have to have training”
*Accommodations for non-drinkers*	
Offering non-alcoholic beverages	“The rule is here that you will never see just a bar set up with only alcohol. We do buffet service of freestanding all night long—water, iced tea”
Not emphasizing alcohol/drinking	“…we never have alcohol front and center”

Results are presented using pseudonyms. Although the results were not shared with participants, they were shared with the campus permitting office and members of the campus’s Healthy Campus organization, both of which provided input on the results’ implications for practice.

## Results

There were 213 alcohol-permitted events. Their characteristics are summarized in Table 3. The number of event guests ranged from 20 to 500, with an average of 71.

**Table 3:** Description of events (N = 213)

Event characteristic	Number of events
Private/public	Private/restricted attendance: 204 Open to public: 9
Guest profile	Faculty, staff, and students only: 39 Faculty and students only: 52 Faculty and staff only: 19 Faculty only: 12 Staff only: 3 Graduate students only: 3 Public: 66 Other: 19
Time	Morning: 9 Afternoon: 174 Evening: 30
Location	On campus: 158 Off campus: 55
Type	Social (e.g., networking and community building): 90 Celebratory (e.g., holiday parties, graduation, welcoming parties, receptions): 63 Work (e.g., gatherings for recruitment interviews, research proposal presentations, symposia, guest lectures, conferences, retreats, and faculty/graduate student orientations): 53 Other (religious services, memorial services, and unknown): 7
Alcohol provided	Wine and/or beer only: 206 Wine, beer, and liquor: 7

### Organizers’ Beliefs About Alcohol

Organizers endorsed the belief that alcohol is needed for a successful event. Fourteen organizers reported that people need alcohol as an incentive to attend events. Christine (61 years old, event specialist) explained that having alcohol at the event “helps people—we found that people will attend knowing there’s going to be alcohol.” Jack (35 years old, assistant professor) said that alcohol encourages graduate student attendance:

“Because it was supposed to be an informal event for them to network with each other and get some practice presenting in front of a non-combative, non-stressful audience. They wanted to provide…a little bit of extra motivation to show up….”

Participants (n = 21) also revealed a belief that people need alcohol to socialize, celebrate, or have fun. Greg (23 years old, events coordinator) stated, “I think they wanted [the event] to be a little more social, and alcohol gets a little more liquid courage”—that is, it reduces social inhibitions. Jeremy (29 years old, graduate student) described alcohol as a “social lubricant,” particularly needed by academics.

“I’m sure you know working with scientists as well, we’re not always the most social bunch. One thing that I’ve always found that would break down some of those socially awkward barriers has been providing something that makes people feel comfortable. For some people, that is alcohol.”

John (29 years old, student services provider) explained, “…to have alcohol kind of [makes it] more of a fun event, rather than all business.” Kayla (26 years old, graduate student) stated, “networking is definitely facilitated by alcohol.”

Participants expressed the idea that people want and request alcohol at events. They reported a belief that these requests should be accommodated. Jim (54 years old, lab research supervisor) said, “Historically, if there is an event that is going to be a social event in our department, faculty likes to bring in wine and beer.” Penelope stated that they offered alcohol at the event because people were, “asking to have alcohol, beer, wine, a nice little selection. A lot of people in our department want it to be there.” In Jeremy’s (29 years old, graduate student) words, “If this is what you guys want, then we want to be accommodating in that respect.” Another idea expressed was that not only do people want alcohol included at events, but also because the attendees are adults, they expect it to be present. Jeremy (29 years old, graduate student) explained:

“There are gonna be adults there. To a certain degree, some people like alcohol. They’re gonna want it at their party…. For those people that do prefer it, it’s there for them.”

Others described providing alcohol as a way to be inclusive. Isaac (35 years old, development director) explained, “having it as an option for those that would like a drink is typically a little more inclusive than not offering it at all.”

### Organizers’ Understanding of Alcohol-Related Risk

Participants’ narratives revealed limited understandings of alcohol risk. The events were characterized by (1) little promotion of responsible drinking, (2) narrow and liability-focused considerations of risk, and (3) an overreliance on informal controls. While the events did not appear to promote irresponsible drinking, most participants (n = 28) indicated that their event included no formal promotion of responsible drinking, such as print flyers or signs encouraging guests to take a ride service in the event of intoxication. When asked about promoting ride service use to prevent post-event drunk driving, Daniel (31 years old, graduate student) said:

“We haven’t really encouraged anything like that. We probably would have a talk with anybody who we could see was becoming inebriated, or maybe a one-on-one. That would be expected. But again, we haven’t seen any sign or need of that. So, we’ve kind of left it to the discretion of the individual to figure out how to manage their inebriation levels.”

Organizers’ considerations of alcohol risk were narrow and liability focused. Some participants emphasized that obtaining the event permit was enough to ensure safety. Peter (33 years old, graduate student) said: “…since we were able to get permits to make it a closed event for us, then it was okay.” Other participants emphasized aspects of the event that they believed made the provision of alcohol less risky. Taylor (59 years old, executive assistant) explained that not allowing hard liquor at the holiday party allowed her to “control the environment.” Other organizers (n = 10) were concerned with event privacy. Morgan (36 years old, administrative officer) explained: “It’s people that we know. We have a list. People check in; so we know who they are. Yeah, it’s usually just a private event. It’s not open; so we’re not giving alcohol to people we don’t know.”

Many participants (n = 25) were primarily concerned with the presence of undergraduate students or people under 21 attending events. Jim (54 years old, lab research supervisor) said, “The main thing is to make sure that underage students don’t get alcohol.” Madison (58 years old, Professor) said, “…we might have an undergrad intern, but I know who they are and we can follow…but if it was a mass number of folks that I don’t know, that are undergraduates, underage, then no, we are not going to [serve alcohol].”

Organizers relied on informal controls in monitoring their events. Some (n = 8) explained that there was always a person tasked with checking guests’ identification to verify that everyone who consumed alcohol was of legal drinking age. However, this person is often someone just “filling in,” not paid to formally perform this job. There was also a reliance on informal monitoring, often by the organizers themselves, of guests’ alcohol consumption. Anna (49 years old, events manager) said, “In the beginning of an event, I’m watching what’s happening at the bar. I’m watching to see who’s lining up, who’s bellying up, who’s ordering what.” She added, “It is constantly something…that I am watching and monitoring and taking care of.” Daniel (31 years old, graduate student) explained, “Especially when you distribute the alcohol, you have to have ideally more than one individual who can be trusted to watch over the distribution of it. And ideally somebody who is assertive enough, that if somebody is taking too much, or if we have reason to think that somebody is underage, that they will actually speak out and talk to them.”

Other organizers (n = 13) relied on the guests themselves to monitor and control their behavior. They tended to believe that the formal promotion of responsible drinking was unnecessary because guests could and should be responsible for monitoring their alcohol consumption and ensuring their own safety. Jack (35 years old, assistant professor) described graduate-student self-regulation: “Responsibility was encouraged by the fact that there was heavy faculty in attendance at the event, and students quite rightfully don’t want to get intoxicated in front of their faculty mentors.”

### Organizers’ Understanding of Addiction and Recovery

Evident in their discussion of accommodations for non-drinkers and their reliance on informal controls, participants had a limited understanding of addiction and recovery. They identified several accommodations for non-drinkers: offering non-alcoholic drink options, keeping alcoholic and non-alcoholic beverages in separate locations, offering food at the event, not over-emphasizing alcohol at the event, and letting participants know ahead of time that there would be alcohol at the event. Most reported that they always served some non-alcoholic beverage. While many (n = 20) reported serving spa water, soft drinks, mocktails or lemonade, others (n = 5) reported that they offered only water, black coffee, or tea as non-alcoholic alternatives. Still others (n = 3) indicated that guests had to provide their own non-alcoholic alternative. Some organizers (n = 13) indicated that their event accommodated non-drinkers by not focusing on or over-emphasizing alcohol or expecting guests to drink alcohol. Jim (54 years old, lab research supervisor) explained, “It’s not like we’re forcing people to drink. It’s just there to take if you want it.” Shelby (50 years old, executive assistant) said:

“We don’t hype up the alcohol consumption. We don’t promote it in any way. People can just go up and select what they want. So I don’t think there’s any kind of pressure at our events for anyone to drink alcohol. Or I don’t believe that anyone would feel weird or different if they didn’t drink it.”

## Discussion

To better understand how higher education institutions can incorporate a culture-of-health approach into existing institutional practices and structures, this study examined alcohol-permitted event organizers’ understandings and management of alcohol risk and accommodation of non-drinking event guests. The organizers endorsed problematic beliefs about alcohol, had limited understandings of alcohol-related risk, addiction, and recovery, and employed a narrow set of strategies and efforts. Thus, there is fertile ground for incorporating a culture-of-health approach into event organization to address alcohol risk and promote inclusion at events with alcohol.

### The Limits of Permits for Events with Alcohol

Our results showed that event permits insufficiently address alcohol-related risks and non-drinkers. We identified various types of alcohol-related risk that are overlooked at events. For example, a reliance on self-control misses the fact that people with substance use disorders are substantially less able to control their behavior (White, 1996). Furthermore, personally knowing the guests does not equate to knowing their recovery status. Given that people with substance use disorders are highly stigmatized (Keyes et al., 2010), they do not always disclose their status. Thus, organizers may misread their guests and, in turn, the level of risk. We also found that organizers give disproportionate attention to drinkers and give less consideration to the safety of other guests, such as people in recovery. These results suggest the possibility that, as has been found in other research (Cleveland et al., 2007), people in recovery may experience alcohol-permitted events to be abstinence hostile or not “sober friendly” and, in turn, either avoid the events or attend but face conditions that threaten their recovery. This study’s results indicate that something in addition to permits is needed to fully address alcohol risk at events.

We also identified little effort by event organizers to promote health—for example, by promoting responsible alcohol consumption or consumption of healthy alternatives to alcohol. Event permits are, by design, focused on protecting the institution from liability; they are not intended to promote health among individuals. For example, participants’ narratives indicated the false belief that safety is achieved by excluding from events members of the community who are less than 21 years old, excluding from alcohol consumption guests who lack identification at the event, or excluding guests who become drunk or disorderly. Permits, thus, do only partial work. Additional work is needed not only to promote greater safety but also to promote health.

### The Potential of a Culture-of-Health Approach to Events with Alcohol

Our results revealed ways that a culture-of-health approach may help to address health and safety at events with alcohol. First, because this approach emphasizes structure over individual agency, it can guide organizers in shaping the event context to influence guest behavior. Rather than relying on individuals to control their behavior, organizers can influence the behavioral choices available to guests. Organizers in the study reported that they monitor closely for the development of problems, such as if a guest becomes drunk and disorderly, but they give little attention to the conditions that could prevent such a development. Unlike the risk management focus of event permits, a culture-of-health approach aims to promote health by creating conditions conducive to healthy behavior. In the case of alcohol, those conditions include what beverage choices are available, how the beverages are displayed, and the extent of emphasis given to alcoholic beverages relative to other beverages.

Second, because a culture-of-health approach emphasizes the collective over individuals, it can guide event organizers in attending to the inclusion of diverse guests—in this case, guests who consume alcohol as well as those who do not. Organizers could be educated about the potential diversity of event guests (drinkers, people in recovery, people who don’t drink for religious or health reasons), conditions that make an event “sober friendly,” and common myths about substance use (e.g., Alcohol always makes events better), substance users and abusers (e.g., People who misuse alcohol simply need more willpower), and substance use recovery (e.g., Water is a sufficient alternative for non-alcohol drinkers). This education could be required as part of event permitting or offered to the university community at large.

Third, the culture-of-health approach emphasizes prevention over intervention. This distinction is evident in cutting-edge approaches to other health and safety issues, such as sexual assault. Research on college sexual assault has highlighted the need to reduce the emphasis on criminal behavior and punishment and instead, promote healthy sexual behavior and community values, such as respect for people’s sexual citizenship (Hirsch & Khan, 2020). In a similar way, event organizers can expand their focus to include not only preventing illegal behavior (e.g., consumption by minors) but also promoting healthy behavior. They can promote safe alcohol consumption and sober post-event driving among drinkers; consumption of healthy beverages, including alternatives to alcohol, among both drinkers and non-drinkers; and recovery maintenance among people in recovery.

### Implications for Prevention and Health Promotion at Events with Alcohol

We identified five culture-of-health considerations for organizers of events with alcohol: beverage choices, reference to beverages, beverage service and placement, safety, and inclusion. Regarding beverage choices, organizers should consider providing creative and tempting non-alcoholic drink options from top-quality non-alcoholic brands that are comparable to alcoholic drink options. They should also consider providing healthy beverages—that is, alternatives to sugar-sweetened or high-calorie beverages.

In terms of how event organizers present and emphasize alcohol, organizers should consider using beverage-neutral language (e.g., “social hour” instead of “happy hour” or “cocktail hour”) in event advertising. This suggestion is consistent with research recommending attention to the ways that events with alcohol are marketed (Holder & Wagenaar, 1994). Event hosts and beverage servers can use beverage-neutral language (e.g., “What can I get you?” instead of “Help yourself to some wine” or “Can I get you another beer?”) at the event. Prior research has shown that server training can reduce guest alcohol consumption and dangerous drinking behavior (e.g., over-drinking and driving under the influence) (Boiler et al., 2011; Jones et al., 2011). Adding to this existing training information about language use might further enhance the prevention outcomes. Finally, event organizers can modify event rituals, replacing traditional ones (e.g., toast involving alcohol) with non-alcoholic ones (e.g., standing ovation).

Regarding beverage service and placement, organizers can provide a non-alcoholic drink menu and an alcoholic drink menu and include on both the drinks’ nutrition content. They can employ identical drinkware to allow non-drinkers to blend in with drinkers. To allow for alcohol-free space, organizers could restrict alcoholic beverage service to a time-limited portion of the event (e.g., social hour or prior to the dinner or ceremony) or to a separate, controlled area (e.g., bar only, indoor vs. outdoor, or designated tables). If providing assigned seating, organizers could ask guests to express their seating preferences (e.g., alcohol-free table or not). These suggestions are consistent with prior research recommending changes to the physical environment to reduce alcohol risk (Gripenberg et al., 2007).

To further address safety, organizers can display information about ride services for guests unable to drive due to alcohol consumption at the event. This guest-targeted intervention may encourage healthy choices for event attendees, but such signage also reduces overserving by alcohol servers (Hughes et al., 2011; Jones et al., 2011).

Regarding inclusion, organizers should ask, what is the purpose of alcohol at the event and is it necessary? Some people may not attend an event because there is alcohol present. If an event is part of a series, organizers should consider including some alcohol-free events in the series to accommodate such people. Organizers can also consider designating and advertising an event as Sober Friendly. Drawing on universal design principles (Burgstahler & Cory, 2010; Steinfeld & Maisel, 2012), a sober-friendly event is intentionally designed to be inclusive of people who do not drink, making requests for special accommodations unnecessary. To avoid a reliance on alcohol consumption to facilitate social interaction, organizers can consider how to encourage people to attend and participate. They can highlight the event’s non-alcoholic benefits (e.g., social networking), and, to facilitate meeting and mingling, provide guest name tags, have icebreakers, and employ a greeter.

While these recommendations could be effective if implemented individually, an optimal strategy would be multi-component. Research has shown that efforts to reduce alcohol-related harm with multiple strategies are markedly more effective than efforts with single strategies (Jones et al., 2011). Such multi-component programs rely on engagement and mobilization from a variety of community members (i.e., event organizers, staff, and attendees) and emphasize a shared responsibility for safety (Jones et al., 2011). The institutional office managing the permitting process could incorporate culture-of-health approaches into its policy and be in charge of promoting them at permitted events. However, other campus units could be engaged to support this effort. For example, if a university has a Healthy Campus organization, it can educate the community about how to have a healthy event with alcohol. Furthermore, employee wellness and student wellness units can participate in promoting culture-of-health approaches at campus events as well as promoting alcohol-free events to demonstrate that alcohol is not necessary to have a successful event. Prior environmental prevention research has shown that alcohol-related policy and education initiatives can be effective (Boiler et al., 2011).

Finally, while the events in this study were not the kind that involve alcohol-industry sponsorship (and in turn, alcohol-promoting signs or novelty items), prior research has documented the role of the alcohol industry in either supporting or interfering with environmental prevention efforts (Begun et al., 2016; Burkhart et al., 2022). Thus, efforts to promote a culture of health should include engagement of the alcohol industry. For example, education about the culture-of-health approach could be required of university beverage vendors.

The implications of this research for prevention science are that infusing a culture of health into events with alcohol has the potential to address both alcohol harm reduction and health promotion, expanding the potential benefits of intervention, including environment prevention efforts.

### Limitations and Future Research

The study was conducted at a single institution. Institutions may vary in terms of their alcohol cultures, event cultures, and event planning procedures, including permitting processes. Future research could explore variation by campus. Our sample included the organizers responsible for obtaining the event permit. Their views may not reflect the views of all people involved in the event planning. For example, administrative staff often executed event plans developed by faculty or administrators. The sample also did not include event guests. Future research, quantitative and qualitative, could examine the views of other people involved in event planning and of event attendees. It could examine reactions to the implementation of a culture-of-health approach to event organization. Prior research on environmental prevention has largely focused on alcohol and nightlife venues as well as on risk reduction (Jones et al., 2011; Hughes et al., 2011; Boiler et al., 2011). Additional research is needed to examine events outside of these venues and health promotion. Such research can determine whether proven effective environmental prevention strategies work at these other events and whether the culture-of-health strategies recommended here, some of which have not yet been studied, are effective in reducing risk and promoting health and inclusion.

## Supplementary Information

Below is the link to the electronic supplementary material.

Supplementary file1 (DOCX 27 kb)
