# Supplementary material for: A Culture of Health and Alcohol-Permitted Events at a U.S. University
Source: J Prev (2022). 2022 Jun 13;43(5):605–22. doi: 10.1007/s10935-022-00686-z (PMC9482600; doi:10.1007/s10935-022-00686-z)
Supplement: Supplementary file 1 — Supplementary file1 (DOCX 27 kb) [file 10935_2022_686_MOESM1_ESM.docx]

Supplementary material

Table 1 Interview Protocol

| **ALCOHOL AT FOCAL EVENT**  1) An alcohol permit was requested for one or more events on _____ titled_____. Was the question of whether to offer alcohol at the event discussed prior to reaching a decision? If so, with whom?  2) How was the decision to offer alcohol at this event reached?  3) What factors influenced the decision?  4) How was the alcohol provided at the event (e.g., free or cash bar; display of alcohol and other drinks)? |
| --- |
| **ACCOMMODATION OF NON-DRINKERS AT FOCAL EVENT**  5) What plans were made to accommodate event guests who are in recovery from alcohol or other drug addiction or who choose not to drink for religious, health, or other reasons? |
| **OTHER EVENTS**  6) If you planned other events with alcohol, other than the focal event, what were they and what factors influenced the decision to offer alcohol at them? Or, if alcohol was not present at the other events, what were the events and what factors influenced the decision not to offer alcohol at them?  7) Are there any special considerations associated with alcohol events in university settings? Please describe.  8) We’ve been talking about events with alcohol and ways to accommodate people who do not drink for various reasons. How, if at all, could accommodations for these people change the events? |
| **ANYTHING ELSE**  9) Is there any other information you wish to share to help us understand how decisions are made about whether to offer alcohol at events or how people who are in recovery or otherwise choose not to drink are accommodated at events? |
| **DEMOGRAPHIC INFORMATION**  10) What is your title?  11) In what unit of the university do you work?  12) What is your gender?  13) What is your age? |
